# Pollen Monitoring by Optical Microscopy and DNA Metabarcoding: Comparative Study and New Insights

**DOI:** 10.3390/ijerph19052624

**Published:** 2022-02-24

**Authors:** Mattia Fragola, Augusto Arsieni, Nicola Carelli, Sabrina Dattoli, Sante Maiellaro, Maria Rita Perrone, Salvatore Romano

**Affiliations:** 1 Department of Mathematics and Physics, University of Salento, 73100 Lecce, Italy; mattia.fragola@unisalento.it (M.F.); mariarita.perrone@unisalento.it (M.R.P.); 2 Azienda Sanitaria Locale (ASL) Brindisi, 72100 Brindisi, Italy; arsienia@tin.it (A.A.); sante.maiellaro@asl.brindisi.it (S.M.); 3 Agenzia Regionale per la Prevenzione e la Protezione dell’Ambiente (ARPA) Puglia, 70126 Bari, Italy; n.carelli@arpa.puglia.it (N.C.); s.dattoli@arpa.puglia.it (S.D.)

**Keywords:** pollen sampling, Hirst-type trap, PM10 sampler, pollen family detection, optical microscopy, DNA metabarcoding approach, pollen family characterization

## Abstract

Environmental samples collected in Brindisi (Italy) by a Hirst-type trap and in Lecce (Italy) by a PM10 sampler were analysed by optical microscopy and DNA-metabarcoding, respectively, to identify airborne pollen and perform an exploratory study, highlighting the benefits and limits of both sampling/detection systems. The Hirst-type trap/optical-microscopy system allowed detecting pollen on average over the full bloom season, since whole pollen grains, whose diameter vary within 10–100 μm, are required for morphological detection with optical microscopy. Conversely, pollen fragments with an aerodynamic diameter ≤10 μm were collected in Lecce by the PM10 sampler. Pollen grains and fragments are spread worldwide by wind/atmospheric turbulences and can age in the atmosphere, but aerial dispersal, aging, and long-range transport of pollen fragments are favoured over those of whole pollen grains because of their smaller size. Twenty-four Streptophyta families were detected in Lecce throughout the sampling year, but only nine out of them were in common with the 21 pollen families identified in Brindisi. Meteorological parameters and advection patterns were rather similar at both study sites, being only 37 km apart in a beeline, but their impact on the sample taxonomic structure was different, likely for the different pollen sampling/detection systems used in the two monitoring areas.

## 1. Introduction

Aerobiology investigates the passive transport of bioaerosols (microorganisms and biological particulate matter) through the air. Plant biodiversity, allergy prevention [1] and gene flow by airborne pollen are only some of its application fields [2]. Pollen is well studied worldwide to investigate the likelihood of human exposure to these aeroallergens, the human sensitivity to them and the severity of allergic symptoms. As a matter of fact, allergic diseases are amongst the most common chronic disorders [3,4], hence the knowledge of atmospheric pollen concentrations in different regions and seasons is compulsory to achieve a better management of the associated diseases [5,6,7,8].

Aeroallergen networks have been globally established in response to the increasing prevalence of pollen allergy and asthma. A typical example is represented by the European Aeroallergen Network (EAN, https://www.ean-net.org/en/ean-studienlandkarte.html, accessed on 21 February 2022) for monitoring pollen and fungal spores in Europe, as well as providing data beyond the continent. The EAN database was founded in 1988 and by early 2019 covered 38 countries and more than 600 measurement sites. POLLnet (http://www.pollnet.it, accessed on 21 February 2022) is the Italian network of SNPA (Sistema Nazionale per la Protezione dell’Ambiente) for the monitoring and study of the biological components of the airborne particulates suspended in the atmosphere, and several of the POLLnet monitoring stations also contributed their measurements to the EAN database.

Routine pollen observations are currently mostly based on morphological analysis by optical microscopy [9,10] as specified by the EN 16868:2019 document. More specifically, a volumetric Hirst-type sampler [11], or a sampler providing comparable data, is suggested in Europe to continuously sample airborne pollen grains and fungal spores in ambient air. Hirst-type impactors capture atmospheric particles on a rotating drum with a silicone-covered tape, in such a way that the particles from a regulated flow of air colliding with the tape remain adhered to the tape. The tape is sent to a laboratory where it is analysed under an optical microscope by trained professionals (e.g., [9]). A high level of training is required for the pollen identification, based on the external morphology of pollen grains, and for many taxa identification below the family level is not possible [12].

Recent technological developments are revolutionising the pollen detection methodologies making real-time high-temporal resolution measurements also possible, as outlined by Clot et al. [8]. They have provided a brief description of the current state of the art, in addition to an overview of new technologies, which have led to the proposals of the EUMETNET AutoPollen network (https://www.eumetnet.eu/eumetnet-and-eu/, accessed on 21 February 2022). Another promising approach is metabarcoding, a DNA-based method for identifying airborne pollen from environmental samples [12,13,14,15] with possible advantages over microscopy methods, even if it cannot provide real-time and high-temporal resolution data. Leontidou et al. [13] amplified a short fragment (about 150 base pairs) of the chloroplast *trnL* gene using universal primers for plants, for the pollen detection from environmental samples. They compared their results with the corresponding ones based on the classical morphological pollen analysis and showed that DNA metabarcoding was efficient and applicable even in complex samples. However, they emphasised the importance of carefully optimising protocols for sample preparation, DNA extraction, and metabarcoding analysis. Swenson and Gemeinholzer [12] have investigated the effects that both the level of exine rupture and lysis incubation time can have on the performance of DNA extraction and Illumina MiSeq sequencing with three common plant DNA barcode regions (*rbcL*, ITS1, and ITS2).

Banchi et al. [15] have recently analysed and discussed the relevance of plant and fungal DNA metabarcoding in aerobiology, since it is considered a powerful, flexible, and reliable method for the estimation of biodiversity in a multiplicity of environmental settings. Gusareva et al. [14] tested the amplification and sequencing of the 16S rRNA gene for bacteria and the 18S rRNA gene for Fungi and Plantae with the main goal being to investigate the microbial communities in the tropical air ecosystems. Another recent study [16] applied the high-throughput sequencing of the 18S rRNA gene to DNA extracts originating from environmental PM10 samples to characterise the seasonal variability of the airborne Fungi and Plantae communities in Lecce (Italy, 40.33° N; 18.11° E). Seven potential pathogenic genera of Fungi in the PM10 samples, in addition to aeroallergens due to the detected most abundant and pervasive Streptophyta genera were detected in [16].

This paper focuses on the characterisation of pollen families sampled and detected in Brindisi by a Hirst-type trap/optical-microscopy system (reference method) and on the characterization of pollen families detected in PM10 samples collected in Lecce, by testing the amplification and sequencing of a region of 18S rRNA gene for Plantae. The main goal of this exploratory study is to compare the contributions of pollen families detected in Lecce (Italy, 40.33° N; 18.11° E) by the DNA-metabarcoding approach with the corresponding ones in samples simultaneously collected in Brindisi (Italy, 40.63° N; 17.94° E) (from July 2018 to June 2019) by a Hirst-type trap/optical-microscopy system. Brindisi and Lecce, which are about 37 km apart in a beeline, are situated in the flat Salento peninsula in south-eastern Italy, therefore it is likely that pollen similarly affects both sites because of their close geographical location and the pollen dispersal by wind/atmospheric turbulence. Brindisi is a monitoring site belonging to AIA (Associazione Italiana Aerobiologia; https://www.ilpolline.it, accessed on 21 February 2022), the first scientific society in Italy dealing with aerobiology, and to the Italian network POLLnet, contributing both to the EAN and ARPA Puglia (https://www.arpa.puglia.it/pagina3409_pollini.html, accessed on 21 February 2022; https://arpapollini.weebly.com/, accessed on 21 February 2022) databases. Hence, the pollen grain sampling and analysis is performed in Brindisi according to the EN 16868:2019 protocol. Lecce is a monitoring site of both the pan-European research infrastructure ACTRIS (https://www.actris.eu, accessed on 21 February 2022) and the related Italian research infrastructure ACTRIS-IT (http://www.actris.it/, accessed on 21 February 2022). The relationships between pollen grain contributions, meteorological parameters, and long-range transported air masses have also been investigated at both sites.

We believe that the results of this exploratory study could contribute to the research activities on the optimisation of the methodologies for sampling and detecting airborne pollen, highlighting, in particular, the benefits and/or limits of the two different sampling/detection systems we have examined here. Our results could also be useful to plan future measurement campaigns and determine the probability of human exposure to aeroallergens, thanks to the main results we have achieved about the impact of meteorological parameters and long-range transported airflows on pollen samples.

## 2. Materials and Methods

### 2.1. Sampling Sites and Pollen Collection

**Figure 1:**
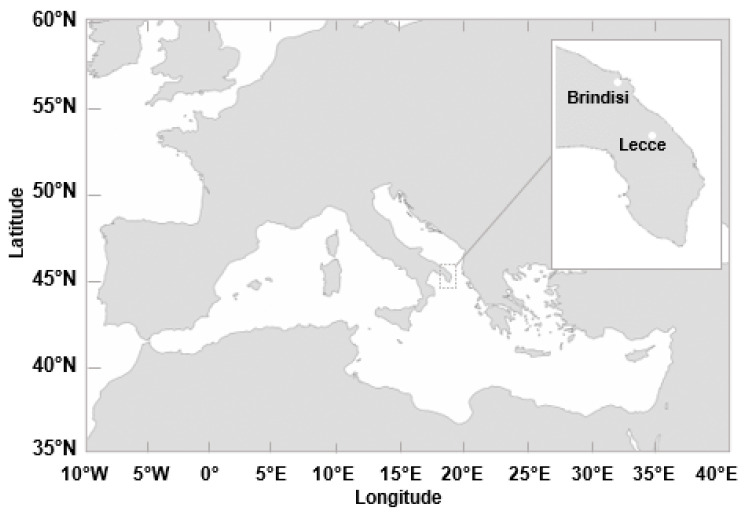
Geographical location of the study area in the Central Mediterranean basin. The location of the pollen monitoring sites both in Brindisi and in Lecce is shown by full dots in the insert.

Measurements in the current study were simultaneously performed in Brindisi and Lecce, which are located in Salento, a flat peninsula at the end of the Apulia region (South-eastern Italy). About 37 km apart, Brindisi overlooks the Adriatic Sea, while Lecce is inland (Figure 1). Environmental samples were collected at the two sites by means of a volumetric and a gravimetric air sampler, respectively. In particular, volumetric air samples were regularly collected in Brindisi by a Hirst-type trap operating at 10 L/min (Lanzoni VPPS 2000, Italy) on the roof (about 20 m AGL) of the Brindisi-ARPA (Regional Agency for the Protection of the Environment) pollen monitoring station (40.63° N; 17.94° E). The EN 16868 (2019) protocol was applied for sampling, handling, identifying, and quantifying pollen in the samples. The air intake slit of the Hirst-type trap is located at the front of the sampler body and is 14 mm (±1 mm) wide by 2 mm (±0.2 mm) high. The sampling belt on the drum is able to sample the air for a maximum time of seven days and the tape is replaced with another blank one at the end of the sampling time interval [9]. Only bioaerosols, such as pollen and other particles from a certain equivalent aerodynamic diameter, cannot follow changes in direction of the air flow and, as a consequence, they collide on the tape surface where they stick.

Gravimetric samplings were performed on the roof of the Mathematics and Physics Department of the University of Salento at about 10 m AGL, which is located in a suburban area in Lecce (40.33° N; 18.11° E). A low volume (38.3 L/min) HYDRA-FAI dual-sampler equipped with a PM10 sampling head, which allows collecting particles and bioaerosols with an aerodynamic diameter ≤10 μm on 47-mm-diameter PTFE (polytetrafluoroethylene) filters, was used. The PTFE filters (TEFLO W/RING 2 μ from VWR International S.R.L.) showed excellent collection efficiency, according to Burton et al. [17]. Thirty-seven PM10 samples were collected from July 2018 to June 2019 by performing 24- or 48-h samplings. We tested different sampling times to investigate the sensitivity of the 18S rRNA metabarcoding analysis for the detection of the airborne eukaryotic communities collected in the sampled PM10 mass, by assuming that the eukaryotic growth or decay was negligible during the sampling time. Each filter was put in a sterile box and stored at −20 °C after sampling, since the eukaryotic growth was unlikely at such a temperature, according to Mykytczuk et al. [18]. Three control filters, which were not subjected to sampling, but handled and stored in the same way as sampled filters, were used as negative control.

### 2.2. Pollen Detection by Morphological Optical Microscopy and DNA Metabarcoding

The pollen-loaded tape of the Hirst-type trap was removed and transferred to the laboratory after each sampling. Then the tape, once cut into daily segments, was mounted on glass slides with fuchsine-stained glycerol jelly and protected with a coverslip, and was analysed with an optical microscope (Olympus BH2, equipped with plan-achromatic optics), after a settling period. Specifically, four transverse transects were read with an immersion 50× optical zoom lens, with the total analysed surface >10%. Pollen grains were identified at a family level and the corresponding count was made. A conversion algorithm could eventually be used to calculate daily concentrations, expressed as pollen grains/m^3^ [9], taking into account that the Hirst-type trap operates at 10 L/min and the sampling time interval.

The PM10-PTFE samples collected in Lecce were firstly processed in aseptic conditions (as described by Romano et al. [19]) to recover environmental DNA. In detail, each filter was cut into 10–15 strips and placed in a 50 mL conical Falcon tube containing a 40 mL phosphate buffer Tween solution (PBT: 0.003% Tween-20, 17 mmol L^−1^ KH_2_PO_4_ and 72 mmol L^−1^ K_2_HPO_4_). The Falcon tube was vortexed for 5 min at maximum power and sonicated at room temperature, then the suspension was poured into a clean Falcon tube. The wash was repeated with an additional 40 mL of PBT to remove any residual material from the filter. Note that both the sample washes were centrifuged for 30 min at 3500× *g* to recover all biological components. The pellets were then processed for DNA extraction using the DNeasy PowerSoil kit (Qiagen, Milan, Italy) and the eluted DNA was precipitated in 10 mM of TrisHCl at pH8, before being sent to Genomix4life S.R.L. (Baronissi, Salerno, Italy) for next-generation sequencing (NGS), quality control, and bioinformatics analyses. Final yield and quality of the extracted DNA were determined using a NanoDrop ND-1000 spectrophotometer (Thermo Scientific, Waltham, MA, USA) and a Qubit Fluorometer 1.0 (Invitrogen Co., Carlsbad, CA, USA). Then, PCR amplification was performed with the primers: NS1: 50-GTAGTCATATGCTTGTCTC-30, and NS2: 50-GGCTGCTGGCACCAGACTTGC-30, which target a region of approximately 515 bp within the 18S rRNA gene (the size of the amplified region plus the primers is approximately 555 bp) [20]. No amplification product was observed in the negative control samples. Each PCR reaction was assembled according to the 18S rDNA Metagenomic Sequencing Library Preparation (Illumina, San Diego, CA, USA) protocol, as described by Fragola et al. [16] and Romano et al. [19]. In particular, the 18S rRNA metagenomics analysis was performed with Kraken, which assigns taxonomic labels to short DNA sequences with high sensitivity and speed, using exact alignments of k-mers and a novel classification algorithm [21]. The database for eukaryotes was composed of RefSeq-complete genomes/proteins [22].

### 2.3. Statistical Analysis and Software for Brindisi and Lecce Samples

Statistical analyses were carried out using the data from all the 37 samples simultaneously collected in Brindisi and Lecce. The biodiversity of the 37 samples was evaluated using the Shannon *H* [23] and Simpson *D* [24] indices. Both parameters have commonly been used to quantify and describe the community (alpha) diversity in different biological samples [16,25,26,27,28]. The Shannon index places a greater weight on species’ richness (total number of present species) [29]. On the other hand, the Simpson index considers species’ evenness (in turn associated with species’ relative abundances) more than species’ richness in its measurement, giving a greater weight to species with a higher frequency in a sample [27,30]. Specifically, Shannon *H* and Simpson *D* indices are calculated as follows:

*H* = −Σ*_i_*
*p_i_* ln *p_i_* (1)

*D* = Σ*_i_* (*p_i_*)^2^ (2)

where *p_i_* is equal to *n_i_/N* for a well-sampled community, with *n_i_* representing the number of individuals in the species *i* and *N* the corresponding total number in the community [29]. *H* tends to increase as the richness of the community increases. *D* represents a measure of dominance, so as if *D* decreases, evenness decreases. The value of *D* ranges between 0 and 1, describing a non-diverse community if *D* = 1 and an infinitely diverse community if *D* = 0.

The one-sample Kolmogorov–Smirnov test (by means of MATLAB kstest function) was applied to verify whether the investigated parameters were not normally distributed. Then, the relationships between pollen contributions and meteorological parameters both for Brindisi and for Lecce samples were investigated using the non-parametric Spearman’s rank-order correlation coefficients, which do not rest upon an assumption of data normality. The PAST (Paleontological Statistics) software package [31] was used to calculate Spearman’s correlation coefficients.

### 2.4. Back-Trajectory Cluster Analysis at the Brindisi and Lecce Monitoring Sites

In order to determine the mean airflows reaching both Brindisi (40.63° N; 17.94° E) and Lecce (40.33° N; 18.11° E) during the investigated period, 4-day analytical air-mass back-trajectories were computed every 1 h for each sampling time interval considered in this study. The back-trajectories at the arrival height of 250 m AGL were used as input for the clustering algorithm, since 250 m AGL represents the minimum starting height for back-trajectory calculation, as recommended in the HYSPLIT version 5 user’s guide (https://www.arl.noaa.gov/documents/reports/hysplit_user_guide.pdf, accessed on 21 February 2022). Note that the HYSPLIT (HYbrid Single-Particle Lagrangian Integrated Trajectory) software version 5.1 (http://ready.arl.noaa.gov/HYSPLIT.php, accessed on 21 February 2022) developed by the NOAA-ARL (National Oceanic and Atmospheric Administration—Air Resources Laboratory) was used for both the back-trajectory and the clustering calculations, along with the meteorological data from NCEP (National Centre for Environmental Prediction). In more detail, we used the meteorological data from NCEP’s twice-daily global analysis at 2.5 degrees resolution, which represents a product of their operational forecast system.

The objective of the back-trajectory cluster analysis is to merge trajectories that are near each other and then represent the identified groups, called clusters, by their mean trajectory. Once the back-trajectories have been assigned to a specific cluster, the algorithm minimises the differences between trajectories within the same cluster and maximises the differences between different clusters. In more detail, the trajectories are combined using an iterative process until the total spatial variance of the individual trajectories with respect to their cluster mean starts to increase. The spatial variance (SV*_i_*_,*j*_) is computed for all endpoints (*k*) of the trajectory (*j*) and its assigned cluster (*i*):

SV*_i_*_,*j*_ *=* Σ*_k_* (P*_j_*_,*k*_ − M*_i_*_,*k*_)^2^ (3)

where P*_j_*_,*k*_ and M*_i_*_,*k*_ are the position vectors for the trajectory *j* and its cluster *i* at the endpoint *k*, respectively, and the sum is taken over the endpoints *k* of the trajectory *j*. Then, the spatial variance of the cluster *i* (CSV*_i_*) is the sum of the spatial variances SV*_i_*_,*j*_ over the total number of the trajectories *j* assigned to each specific cluster *i*:

CSV*_i_* = Σ*_j_* SV*_i_*_,*j*_ (4)

The total spatial variance (TSV) is the sum of CSV*_i_* over all clusters *i*:

TSV = Σ*_i_* CSV*_i_* (5)

## 3. Results and Discussion

### 3.1. Characterisation of Meteorological Parameters and Pollen Families in Brindisi

Pollen samples collected in Brindisi (Figure 1) are analysed in this subsection, whose results are based on optical microscopy. Table 1 provides the sampling date and sampling time (ST) interval of the 37 samples collected from July 2018 to June 2019. Samples are listed in Table 1 according to the sampling month by assuming S1 collected on 7 January 2019 as the first sample. Note that N° Tot. Pollen Grains in Table 1 provides for each sample the number of pollen grains associated with the families monitored at Brindisi according to AIA protocol (https://www.ilpolline.it/wp-content/uploads/2019/05/Regolamento-della-Rete-Italiana-di-Monitoraggio-in-Aerobiologia.pdf, accessed on 21 February 2022), in addition to the number of pollen grains due to *Others* and *Unclassified* defined in the following. The Shannon and Simpson index values at the family level are also provided in Table 1, which have been calculated by using the N° Tot. Pollen Grains values associated with each sample. In addition to Sampling Date and ST intervals, Table S1 provides for each sample the mean values of meteorological parameters (except Cumulative Rain, for which the total sum is reported), calculated according to the corresponding ST of Brindisi and Lecce. The reported values of Temperature (T), Relative Humidity (RH), Pressure (P), Cumulative Rain (CR), Wind Speed (WS), and Wind Direction (WD) have been calculated from the corresponding 30-min data provided by ARPA (Agenzia Regionale per la Protezione dell’Ambiente) Puglia for both sites (https://www.arpa.puglia.it/pagina2839_meteo.html, accessed on 20 October 2021). More specifically, the mean values of T, RH, P, and WS reported in Table S1 provide the arithmetic means of the 30-min values provided by ARPA Puglia for the ST intervals, while CR values are the total amount of rain in the ST interval. Note that the WD mean values provide the “unit-vector mean wind direction”, according to the EPA-454/R-99-005 Meteorological Monitoring Guidance [32]. Meteorological parameter values and sample-by-sample meteorological parameter changes were very similar at Lecce and Brindisi, being only 37 km apart (Table S1). In particular, the mean difference between the T, RH, P, CR, and WS values in Brindisi and in Lecce were equal to 0.4 °C, 2%, 4.8 mbar, 1.6 mm, and 0.1 ms^−1^, respectively. Meteorological parameter values for winter (January, February, March), spring (April, May, June), summer (July, August, September), and autumn (October, November, December) are in Table S2, to further show the meteorological parameter similarity between the two analysed sites. Largest T and smallest RH values were reached at both sites in summer, while largest CR and WS values were reached in autumn and in spring, respectively.

**Figure 2:**
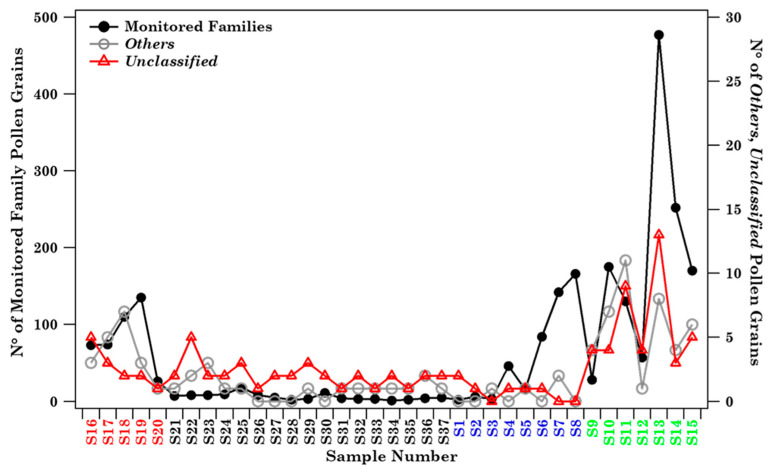
Total number of pollen grains per sample of the 21 allergenic pollen families monitored in Brindisi (black full dots). Open dots and triangles show the number of pollen grains due to *Others* and *Unclassified* contributions per sample. Samples are listed according to the sampling date. Each sample colour indicates a different season (red: summer, black: autumn, blue: winter, green: spring). Solid lines join the discrete data points to better visualise their respective time evolution.

Thirty-six different pollen families are regularly monitored at Brindisi by morphological analysis with optical microscopy, according to the EAN, POLLnet, and AIA protocols. Twenty-three out of the thirty-six different pollen families are listed in Table S3. Apart from Papaveraceae and Vitaceae, which were only detected at Lecce during the monitoring campaign of this study, all the other 21 pollen families were detected in one or more of the Brindisi samples. Table S3 also shows the pollen grain number per family detected in each sample, in addition to the *Others* and *Unclassified* contributions. *Others* represents the grain number per sample of the identified pollen families not included within the 23-family list of Table S3, while *Unclassified* represents the unclassified pollen. The Relative Abundance (RA) percentage of the family pollen grains detected in each sample is reported in Table S4. Bold-marked pollen families in Tables S3 and S4 represent the 15 (out of 16) pollen families regularly monitored at Brindisi according to EAN and POLLnet (http://www.pollnet.it, accessed on 21 February 2022) protocols. Figure S1 shows by a colour map the mean monthly concentration of the 16 pollen families monitored at Brindisi according to the POLLnet protocol (http://www.pollnet.it/, accessed on 21 February 2022), to highlight their main blooming months. It is based on data collected at Brindisi from 2013 to 2015. Note that the eight not-bold-marked pollen families reported in Table S3 are also monitored at Brindisi and considered as aeroallergens, according to the AIA protocol. Figure 2 shows the total number of pollen grains (black full dots) due to all the families detected in each sample, in addition to the *Others* and *Unclassified* contribution per sample, to display the seasonal dependence of the detected pollen grains. Samples are listed according to the sampling time, starting from sample S16 collected on 4 July 2018.

**Figure 3:**
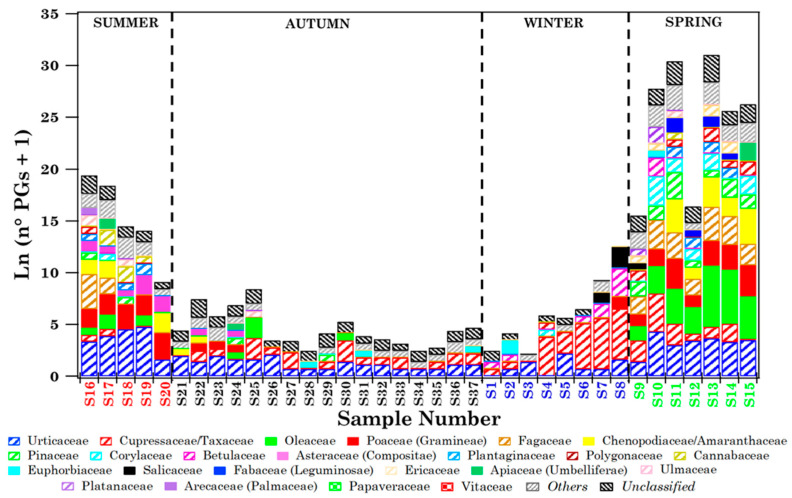
Natural logarithm of the number of pollen grains (n° PGs) + 1, associated with the 21 identified families in each sample collected at Brindisi, in addition to the *Others* and *Unclassified* pollen grains. Samples are listed according to the sampling date. Each sample colour indicates a different season (red: summer, black: autumn, blue: winter, green: spring).

The pollen grain number was larger in spring and summer samples than in autumn and winter ones, likely because spring and summer are the blossoming seasons of most of the detected pollen families, according to Figure S1. *Others* and *Unclassified* contributions follow a similar trend. Figure 3 shows, by coloured bars, the natural logarithm of the number of pollen grains (+1) associated with the 21 identified families in each sample collected at Brindisi, in addition to the *Others* and *Unclassified* grain numbers, to better visualise the contribution of the less abundant components. The corresponding RA percentages per sample are in Figure S2.

**Figure 4:**
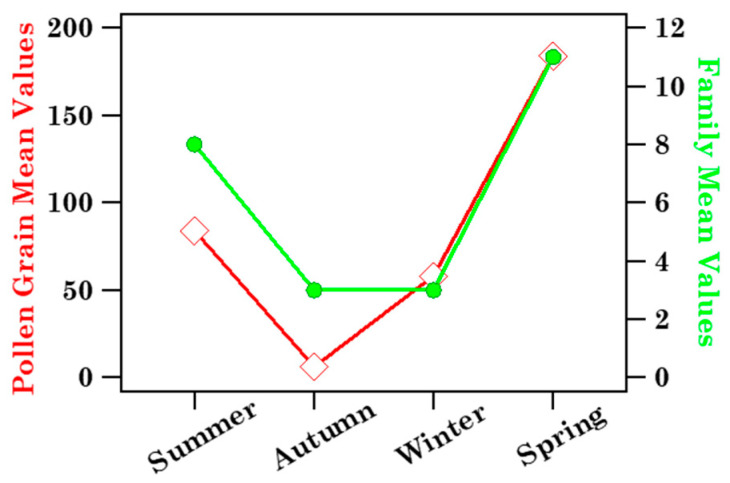
Mean values of the number of pollen grains and families monitored in Brindisi across the four seasons: summer (July, August, September), autumn (October, November, December), winter (January, February, March), and spring (April, May, June). Solid lines join the discrete data points to better visualize the respective seasonal trend. Seasons are listed according to the sampling date.

Both Figure 3 and Figure S2 provide a clear overview of the taxonomic composition of each sample, as well as the day-by-day and seasonal taxonomic variations of the samples. Black dotted vertical lines in Figure 3 and Figure S2 identify the samples collected in different seasons: summer (S16–S20), autumn (S21–S37), winter (S1–S8), and spring (S9–S15). More specifically, Figure 3 shows that the contribution per sample of the most abundant pollen was also larger in spring and summer than in autumn and winter. Figure 4 displays the mean value of the total number of grains (diamonds) and families (full dots) associated with each season.

The rather high CR values in autumn and winter (Table S2), in addition to high RH values, which may have fostered the swelling and rupture of pollen grains [7,33], preventing their morphological identification by optical microscopy, have likely contributed to their low autumn and winter mean values, in addition to the small number of pollen families blooming in autumn and winter (Figure S1). Oleaceae pollen grains were the most abundant and have mainly been detected in spring samples up to 371 grains/sample (Figure 3 and Table S3), in good accordance with Figure S1. Olive cultivation is widespread in southern Europe and North Africa [9] and, consequently, at the study sites. Urticaceae, the second most abundant pollen family (Table S3), were detected in all samples (Figure 3), except for samples S1 and S4, which were collected in January. Large Urticaceae grain numbers, up to 118 grains/sample (Table S3), have only been found in the samples collected in April, May, and July, in good accordance with the monthly evolution of the Urticaceae pollen concentration reported in Figure S1. Urticaceae is a pollen family distributed all over the world, apart from polar regions [5]. Cupressaceae/Taxaceae were the third most abundant pollen families in the Brindisi samples (Table S3). The Cupressaceae and Taxaceae pollen grains have a quite similar morphological structure, therefore they cannot be distinguished by optical microscopy and are coupled in this study. Cupressaceae/Taxaceae have been detected in most of the samples, but quite high numbers of pollen grains have been observed mainly in February (Figure S1 and Table S3), as reported in Porto (Portugal) by Gomes et al. [7]. Moreover, Cupressaceae pollen grains have a tendency to break apart. Indeed, Rezanejad [34] and Shahali et al. [35] observed in urban areas that the interaction between Cupressaceae pollen and atmospheric pollution could increase the fragility of the pollen grain exine, causing several collapses and cracks and facilitating its break.

#### 3.1.1. Pollen Grain Concentration Relationships with Meteorological Parameters in Brindisi

The impact of T, RH, P, and WS mean values per sample on the pollen grain concentration per sample associated with each detected family, *Others* and *Unclassified* pollen concentration per sample, and the total pollen grain number (n° PGs) concentration per sample is investigated in this subsection to contribute to a better understanding of Figure 3 results. Only the five pollen families detected at least in 30% of the samples (Cupressaceae/Taxaceae, Oleaceae, Pinaceae, Poaceae, and Urticaceae) have been considered for the correlation analysis. Table S5 displays the Spearman correlation coefficients, where those significant at a *p*-level < 0.05 and 0.01 are marked by * and **, respectively. Negative correlation coefficients are marked in bold. Significant correlation coefficients with meteorological parameters are summarised in Table 2, in which parameters with negative correlation coefficients are in bold. Apart from Cupressaceae/Taxaceae, the other four pollen families were significantly and positively correlated with T. *Others* and n° PGs were also positively correlated with T. These results indicate that T was the main factor affecting the pollen concentration in the atmosphere and/or allowing for the collection of pollen grains by the Hirst-type impactor.

No pollen family was significantly correlated with RH in Brindisi, in contrast to some previous studies (e.g., [7]). The small variability range of RH within the 37 sampling days of this study (Table S1) has likely contributed to the above results. No pollen family was significantly correlated with WS, in accordance with previous studies [7], likely because the Hirst-type impactor sampling system allows collecting only pollen grains that cannot adapt to the change in the air flow direction. However, the small WS variability range within the 37 sampling days of this study (Table S1) may also have contributed to this last result. Indeed, only a few days were characterised by WS > 3 m s^−^^1^. Pressure was positively correlated with Cupressaceae/Taxaceae and anti-correlated with Pinaceae. No significant correlations between meteorological parameters were found. Table S6 summarises the significant correlations between the five pollen families and with n° PGs. Apart from Cupressaceae/Taxaceae, which were only positively correlated with n° PGs, the other four pollen families were positively intercorrelated and positively correlated with n° PGs, *Others*, and *Unclassified*.

#### 3.1.2. Biodiversity of Brindisi Samples and Analysis of Case Studies

The Shannon (*H*) and Simpson (*D*) indices at the family level have been calculated to characterise the biodiversity of the 37 collected samples [36]. Note that both the *H* and *D* indices are highly variable since in the short term they are strictly related to the abundance of the airborne pollen from different families, which in turn varies unevenly. In fact, Pace et al. [37] observed that different taxa produce different quantities of pollen and are differently influenced by meteorological conditions, and these aspects can present a significant effect on the most common biodiversity indices. *H* and *D* values are displayed in Table 1, which shows that *H* and *D* reached the highest value (1.98) and one of the smallest values (0.11), respectively, in sample S11 where 13 different pollen families were identified (Figure 3). Sample 11 was collected on 9 May 2019 and most of the monitored families reached high concentrations in May, according to Figure S1. The pathways of the four-day analytical back-trajectories that reached Brindisi at 250, 500, and 750 m Above Ground Level (AGL) on 9 May 2019 at 12:00 UTC are plotted in Figure S3a to likely infer the contribution of long-range-transported air masses. Figure S3a shows that air masses crossed northern European countries, Italy and the Tyrrhenian and Ionian Seas at very low altitudes before reaching Brindisi at 12:00 UTC. Therefore, long-range transported airflows may have likely favoured the advection of pollen grains in Brindisi. The mean wind speed at the surface (4 m s^−1^) reached one of the highest values on the 9th of May and the wind direction from the southeast was in reasonable accordance with the back-trajectory pathways at the arrival time. The taxonomic profile of sample S11 (Table S3 and Figure 3) shows that Pinaceae reached the highest number of pollen grains in that sample, likely for long-range transported contributions, as Pinaceae is widespread all over southern Europe.

The number of pollen grains, which was equal to 130 in sample S11, reached the highest value (477) in sample S13, collected on 23 May 2019 (Table 1). In this case, most of the pollen grains were due to the Oleaceae (Table S3) and, because of the predominance of this family, *H* and *D* were equal to 0.93 and 0.53, respectively (Table 1). Figure S3b shows the four-day analytical back-trajectories that reached Brindisi on 23 May 2019 at 12:00 UTC. Air masses that crossed the Tyrrhenian Sea, Central Italy, and the Adriatic Sea at very low altitudes before reaching Brindisi likely contributed to the massive advection of Oleaceae pollen grains on that day. In fact, olive cultivation extends throughout the countries overlooking the Mediterranean basin, according to Negral et al. [9]. The mean wind speed at the surface was equal to 1.4 m s^−1^ on the 23rd of May and the wind direction from North-West-North was in reasonable accordance with the back-trajectory pathways at the arrival time.

**Figure 5:**
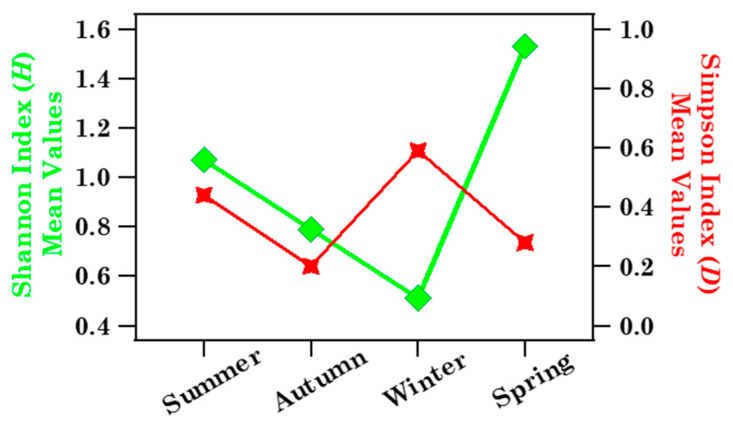
Brindisi Shannon and Simpson index mean values at the family level across the four seasons: summer (July, August, September), autumn (October, November, December), winter (January, February, March), and spring (April, May, June). Solid lines join the discrete data points to better visualise their respective seasonal trend.

Note that only Urticaceae pollen grains were identified in samples S3 and S34 (Figure 3). CR reached the largest values during S3 (14 mm) and S34 (12.8 mm) sampling time and the RH mean values were equal to 86 and 87% over the S3 and S34 sampling, respectively (Table S1). As previously mentioned, pollen grains may absorb moisture from the air and swell; then, especially in case of some heavy rain event, such as during or right after thunderstorms, swelling may cause the grains to break apart making their identification by optical microscopy difficult. Hence, the exclusive detection of Urticaceae pollen in samples S3 and S34 could indicate that these grains have a weak tendency to swell and break under high RH and CR levels, in contrast to Cupressaceae [7]. In S3, the *H* and *D* values were equal to 0.22 and 0.56, respectively, and the corresponding values of sample S34 were 0.35 and 0.06, respectively, since only the Urticaceae pollen grains were detected in both samples. Figure 5 displays the seasonal dependence of *H* (diamonds) and *D* (stars) mean values, revealing that *H* reached the highest value in spring and the smallest in winter, while *D* was smallest in autumn and highest in winter.

#### 3.1.3. Impact of Advection Patterns on Pollen Grain Numbers and Families in Brindisi

**Figure 6:**
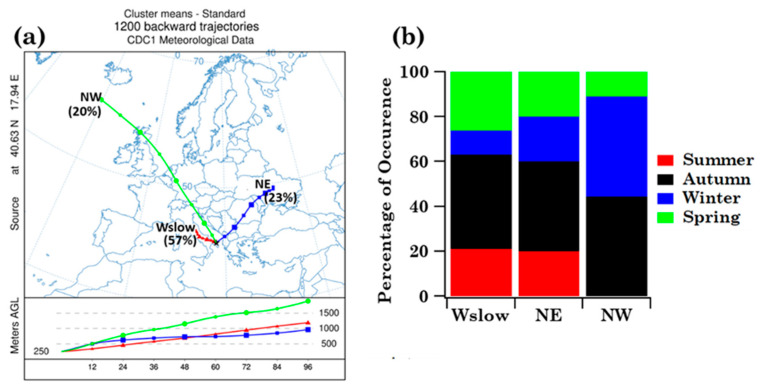
(**a**) Centroids associated with the main airflow types calculated by the cluster analysis of the four-day analytical back-trajectories that reached Brindisi at 250 m AGL throughout the sampling period and (**b**) the corresponding airflow frequency of occurrence in summer, autumn, winter, and spring in Brindisi.

The main airflow pathways in Brindisi have been computed to evaluate the likely impact of long-range transported air masses on pollen grains and families, which some case studies in the previous subsection have highlighted. The cluster analysis of the four-day analytical back-trajectories that reached Brindisi at 250 m AGL throughout the sampling period was performed according to the procedure outlined in Section 2.4 and led to the identification of three main air-flow types, which are represented in Figure 6a by their centroids. Airflow types, labelled according to their overall direction and length, are North-East (NE), North-West (NW), and West-slow (Wslow). Short trajectories are indicative of slow-moving air masses, while extremely long trajectories are indicative of fast flows [38].

**Figure 7:**
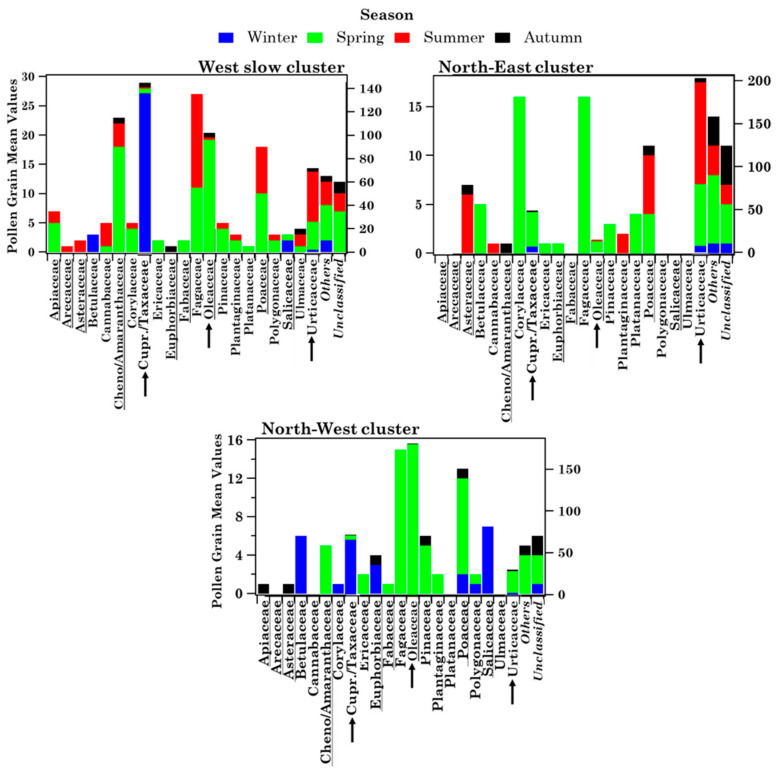
Seasonal dependence (by stacked bar plots) of the mean pollen grain number from each of the 21 detected pollen families, *Others* and *Unclassified* for the three main advection patterns identified in Brindisi. The right scale refers to the families indicated by the black arrows. Each colour indicates a season, while the height of each colour bar represents the mean value of pollen grains. The underlined pollen families have also been detected in Lecce.

Wslow flows, which were the most frequent (57%) according to the percentage of occurrence shown in Figure 6a, crossed several central Italy regions at altitudes below 1000 m AGL in the last 72 h before reaching Brindisi. The NE advection pattern, which crossed several eastern European countries at altitudes smaller than 1000 m before reaching Brindisi, was the second most abundant (23%) airflow. The frequency of occurrence of the NW pattern was equal to 20% and its airflows crossed the Atlantic and several northern European countries at altitudes beyond 1000 m before crossing the Adriatic Sea and reaching Brindisi. Figure 6b shows the seasonal frequency of occurrence of the airflow types. Wslow airflows were the most abundant in autumn and least abundant in winter, during the sampling period of this study. The frequency of occurrence of NE airflows was similar to the Wslow one, while NW airflows were prevailing in autumn and winter and were not detected in summer during the monitoring days. Mean values ± the standard error of the mean (SEM), and minimum (Min) and maximum (Max) values of family number, family pollen grains, *Others* and *Unclassified* pollen grains, as well as Shannon and Simpson indices, are shown in Table 3 to characterise the main features of the samples associated with each cluster. As expected, the largest mean values of pollen family and grain numbers and of *H* were associated with Wslow airflows, which were also the most frequent (57%), while the smallest mean values were associated with the least frequent (20%) NE airflows. The taxonomic structure of the samples associated with each cluster is in Figure S4. Each sample has been assigned to a trajectory cluster if at least three of the four-day back-trajectories belonged to that cluster during each sampling day. Consequently, samples S1, S29, S35, and S36 have not been assigned to any cluster. Figure S4 indicates that Cupressaceae/Taxaceae and Euphorbiaceae likely reached the highest RAs in the samples collected during NW airflows. However, the availability of few samples did not allow performing a robust statistical analysis. Figure 7 displays, for each detected family, the mean values per season of the family grain number, *Others*, and *Unclassified* contributing to each cluster. The right-hand scale in Figure 7 refers to the pollen families indicated by the black arrows, to better visualise their contributions. The pollen grain number of each detected family per season is shown in Table S7 for the three back-trajectory clusters.

All the 21 pollen families were detected during Wslow airflows, likely because they are common on the territory of southern Italy crossed by these airflows, but also because Wslow airflows were the most frequent. Most of the 21 families were detected in different seasons, except for Arecaceae, which were found only in summer, likely because they grow mostly in warm and tropical regions. About 70% of 21 pollen families have been identified during NW and NE airflows and most of them mainly in spring. Cupressaceae/Taxaceae pollen reached the largest contribution in winter during Wslow and NW airflows, as winter is their blooming season (Figure S1), and in spring during NE airflows, likely because of the contribution of long-range transported air masses. Oleaceae and Poaceae were also detected in different seasons within the clusters, but their mean contribution was largest in spring during Wslow and NW airflows (Figure 7). Fagaceae, whose blossoming season is spring (Figure S1), reached the largest contribution in that season during Wslow, NE, and NW airflows.

### 3.2. Streptophyta Family Characterisation in Lecce by DNA-Metabarcoding Approach

**Figure 8:**
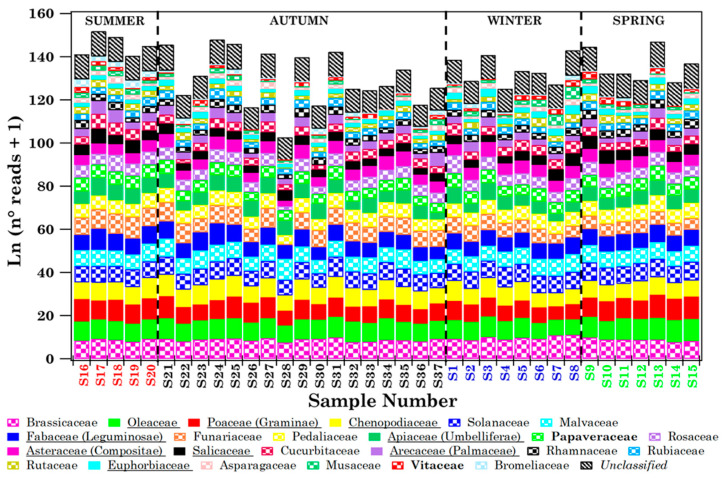
Natural logarithm of the number of reads (+1) of the Streptophyta families in each of the 37 samples collected in Lecce. The detected 24 Streptophyta families are listed in the legend, where the underlined ones represent the nine pollen families detected also in Brindisi. Papaveraceae and Vitaceae, which are in bold, have only been detected in Lecce. The not-classified Streptophyta families, denoted as *Unclassified*, are also reported in the plot. Samples are listed according to the sampling date. Each sample colour indicates a different season (red: summer, black: autumn, blue: winter, and green: spring).

The Streptophyta families detected in each of the 37 samples collected in Lecce (Figure 1) from July 2018 to June 2019 are analysed in this subsection, so as to show similarities and differences from the pollen families detected in Brindisi. The 18S rRNA gene partial sequencing was used to detect Streptophyta genera in PM10 samples [16], as mentioned. Twenty-four Streptophyta families, which are all potential sources of aeroallergens, except for Funariaceae (according to the AllerBase Allergen Database [39,40]), have been detected in the Lecce samples in the current study. The natural logarithm of the read number (+1) of the 24 detected Streptophyta families is shown in Figure 8 by different colours/symbols to visualise their contributions in the 37 samples. The RA% of the 24 Streptophyta families in the 37 samples is shown in Figure S5.

Table 4 displays the sequence-read total number due to the Streptophyta families (also considering the *Unclassified* contribution) and the total number of families detected in each of the 37 samples, in addition to *H* and *D* values at the family level. It shows that the number of detected pollen families per sample varied from 19 to 24. The read number of each Streptophyta family and the *Unclassified* reads in each of the 37 samples are given in Table S8. Only 11 out of the 24 families listed in Table S8 are part of the list of pollen families, regularly monitored in Brindisi (Table S3) according to the AIA protocol. Papaveraceae and Vitaceae pollen families, which are widespread over south-eastern Italy, were not revealed by optical microscopy in Brindisi throughout the sampling time of this study, while in Lecce Papaveraceae were detected in all samples and Vitaceae were detected in 28 out of 37 samples. Therefore, only nine out of the twenty-four pollen families detected in Lecce are shared with Brindisi, and they are underlined in the legend of Figure 8. Colours/symbols associated with the nine families shared between the two sites are equal in Figure 3 and Figure 8, to better visualise their contributions at both sites. These families have been identified in all samples in Lecce, whilst in Brindisi they were detected mainly in spring samples (Figure 3).

The particulate sampling/detection system used in Lecce has likely contributed to the detection of only nine pollen families shared with Brindisi. In particular, the PM10 sampler allows collecting only pollen grains and/or fragments with an aerodynamic diameter ≤10 μm in Lecce, while whole pollen, whose grain diameter ranges from about 10 to over 100 μm, is required in Brindisi for the morphological detection by optical microscopy. Therefore, pollen grains and/or fragments with an aerodynamic diameter >10 μm have not been detected by the PM10 sampler in Lecce. As an example, Urticaceae, the second most abundant pollen family in Brindisi (Figure 3), were not identified in any Lecce sample. The size of the Urticaceae pollen grains (14–19 μm in diameter; https://www.arpae.it/it/temi-ambientali/pollini/schede-botaniche/urticacee, accessed on 21 February 2022) and/or the weak tendency of the pollen wall exine-layer to break into fragments ≤10 μm, releasing its cytoplasmic content, have likely contributed to this result. Cupressaceae/Taxaceae, the third most abundant pollen family in Brindisi, have also not been detected in Lecce, even if the exine layer of the Cupressaceae pollen walls is known to be very thin and easy to break, causing the release of the pollen protoplast due to pollen bursting [41]. However, although some DNA extracts displayed a positive correlation between increased rupture and DNA yield, Swenson and Gemeinholzer [12] reported that an increasing time of lysis incubation was associated with a decreased DNA yield. Therefore, in addition to the PM10 sampler used in Lecce, the structure/properties of the pollen fragments, the DNA extraction protocol, and the successive 18S rRNA gene partial sequencing may have likely contributed to the detection of only nine out of the twenty-one pollen families detected in Brindisi [15]. In particular, the DNA extraction could greatly influence the DNA yield of thin-walled pollen grains that are potentially more susceptible to lysis than thick-walled species, according to Swenson and Gemeinholzer [12]. Work is currently in progress in Lecce to optimise the pollen sampling system, as well as the DNA extraction procedure and the DNA sequencing from environmental samples.

**Figure 9:**
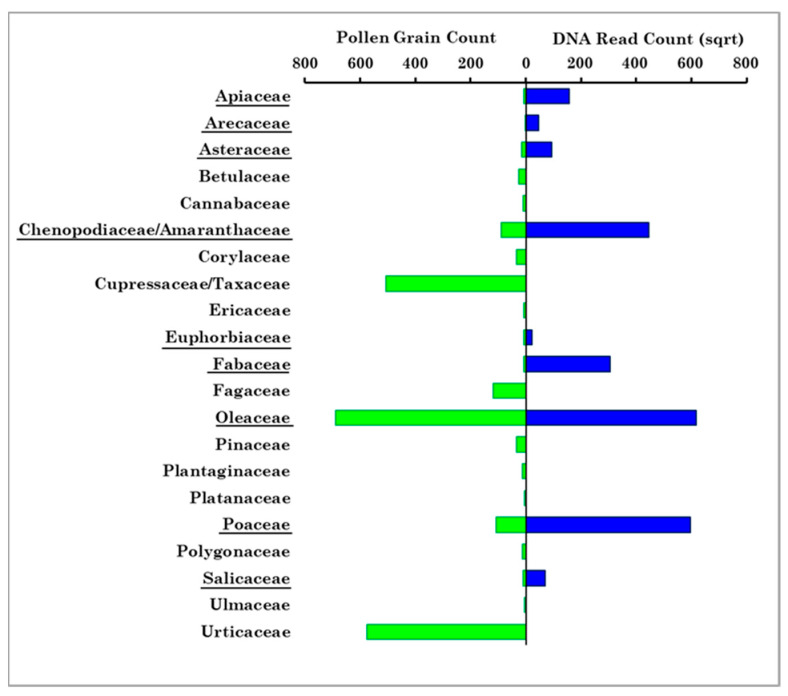
Comparison between the total number of each of the twenty-one family pollen grains counted by optical microscopy in Brindisi (Table S3) and the corresponding number of sequence reads of the nine shared pollen families detected in Lecce, which are underlined in the figure. The number of sequence reads has been square-root-transformed for visualisation purposes.

Figure 9 displays the number of pollen grains for each of the 21 families detected in Brindisi and the square root of the total number of reads for the nine Streptophyta families shared with Brindisi. Previous studies (e.g., [42]) have shown that the number of sequence reads associated with each identified family is generally closely correlated with the number of pollen grains identified by optical microscopy, even if many factors associated with handling samples, technical processes, or the biological material itself can affect the accuracy of metabarcoding quantification. The Hirst technique, which may have a significant uncertainty at counts below 20 pollen grains/m^3^, according to Gottardini et al. [43], Šikoparija et al. [44], and Adamov et al. [45], may have also contributed to the factors accounting for the differences between Brindisi and Lecce results. Campbell et al. [46] also found that the number of pollen grains counted microscopically emulated DNA read counts, even if there were some observable differences.

**Figure 10:**
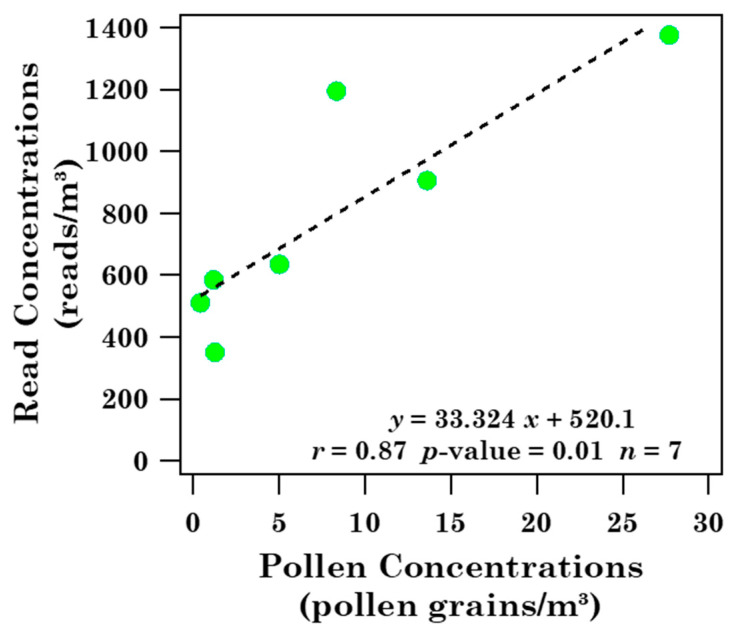
Read concentration as a function of the corresponding pollen grain concentration of the common pollen families detected both in Brindisi and in Lecce in spring. Fitting regression line equation, Pearson correlation coefficient (*r*), *p*-value, and the number of points (*n*) are also given.

Figure 10 shows the read concentration (expressed as reads/m^3^) as a function of the corresponding pollen grain concentration (expressed as grains/m^3^) per sample due to the shared families in spring samples (S9–S15). The fitting regression line equation is also provided in addition to the Pearson correlation coefficient (*r* = 0.87), which indicates that data points are linearly correlated at a *p*-level ≤ 0.01. The analysis has been restricted to the spring samples to limit the uncertainties associated with low grain numbers, according to the above comments [43,44,45]. In fact, higher grain numbers have on average been found only in the spring samples (Table 1). We believe that these last results support the comparison between the pollen families detected in Brindisi by optical microscopy and the ones detected in Lecce by 18S rRNA gene partial sequencing.

**Figure 11:**
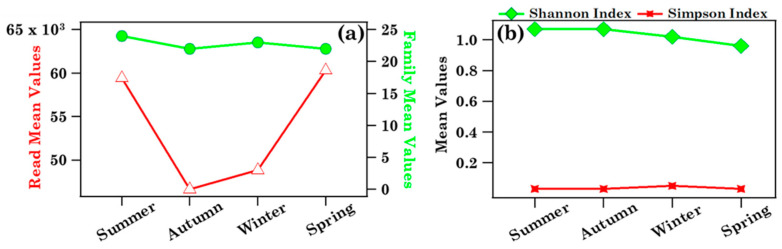
Mean values of (**a**) the number of Streptophyta reads (open red triangles) and families (full green dots) and (**b**) Shannon (green diamonds) and Simpson (red stars) indices at the family level in Lecce across the four seasons: summer (July, August, September), autumn (October, November, December), winter (January, February, March), and spring (April, May, June). Solid lines join the discrete data points to better visualise their respective seasonal trend.

The seasonal dependence of Streptophyta reads and family number mean values, calculated from Table 4, is plotted in Figure 11a. Table 4 shows that *D* and *H* values varied within the 0.71–1.19 and 0.02–0.12 range, respectively. The seasonal dependence of the respective mean values is shown in Figure 11b. Streptophyta reads reached the largest value in spring in reasonable accordance with the pollen grain number trend (Figure 4), while family number and *H* and *D* values did not show any significant seasonal dependence, likely for the contribution of aged and/or long-range transported pollen fragments at the study site over the sampling period.

#### 3.2.1. Impact of Meteorological Parameters and Advection Patterns on the Pollen Families Detected in Lecce

Spearman correlation coefficients between meteorological parameters and the 24 Streptophyta family, *Unclassified*, and total read number concentrations are shown in Table S9, where the nine pollen families shared with Brindisi are underlined. Several Streptophyta families were positively or negatively intercorrelated. In particular, Oleaceae were positively correlated with Poaceae, as found in Brindisi (Tables S5 and S6). The significant Spearman correlation coefficients between meteorological parameters and the nine families shared with Brindisi are summarised in Table 2, where negative correlation coefficients are bold-marked. Poaceae were the only family correlated with T at both sites, while five (Asteraceae, Brassicaceae, Cucurbitaceae, Euphorbiaceae, and Solanaceae) out of the 24 pollen families detected in Lecce were negatively correlated with T (Table S9). Cucurbitaceae, Papaveraceae, and Rosaceae were negatively correlated with RH. Oleaceae and Rubiaceae were also negatively correlated with P, while Fabaceae, Papaveraceae, Poaceae, Rosaceae, and Salicaceae were positively correlated with WS, which in turn was negatively correlated with RH (Table 2). The different sampling/detection systems used in Brindisi and in Lecce could be the main responsible for the observed differences.

**Figure 12:**
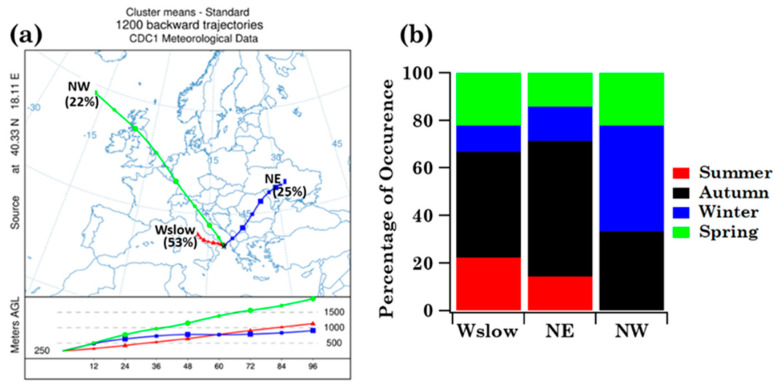
(**a**) Centroids associated with the main airflow types calculated by the cluster analysis of the four-day analytical back-trajectories that reached Lecce at 250 m AGL throughout the sampling period and (**b**) corresponding airflow frequency of occurrence in summer, autumn, winter, and spring in Lecce.

**Figure 13:**
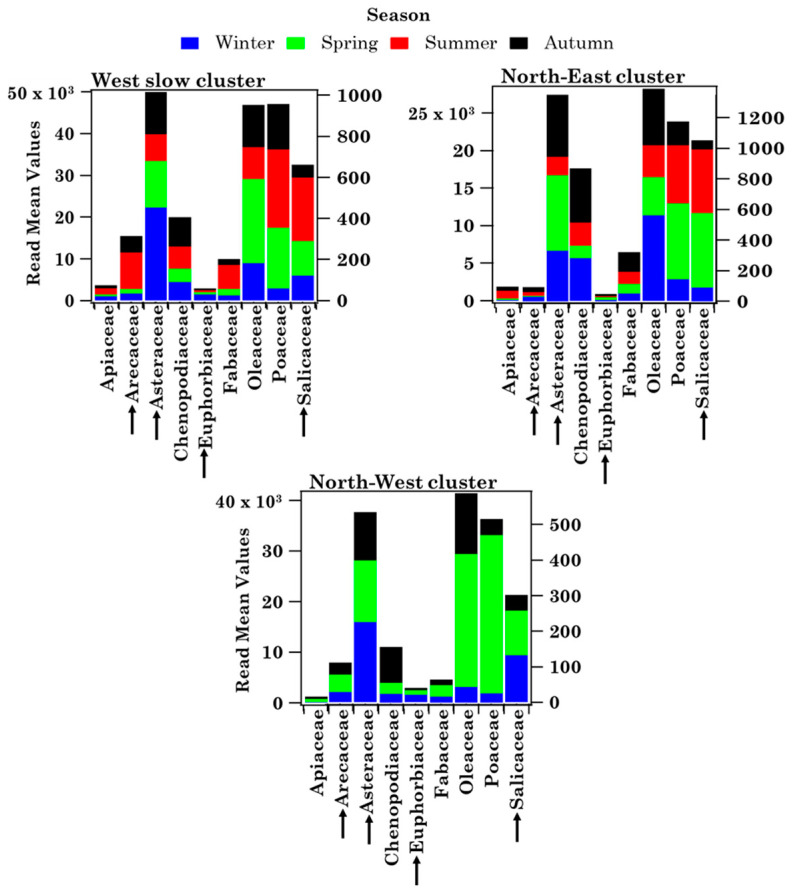
Read mean values (per season) of the nine families shared between Brindisi and Lecce. Each colour indicates a season, while the height of each colour bar represents the read mean value per season. The right scale refers to the families indicated by the black arrows.

The main airflow pathways in Lecce, which have been computed by the analytical back-trajectory cluster analysis, are shown in Figure 12a and they are rather similar to the ones in Brindisi (Figure 6a). The seasonal dependence of their frequency of occurrence is in Figure 12b and it is also quite similar to Brindisi mainly during Wslow airflows, likely because they were the most frequent at both sites. In fact, eighteen, seven, and nine samples are associated with the Wslow, NE, and NW cluster, respectively (Table S10). Figure 13 displays the seasonal mean read values of the nine shared pollen families contributing to each back-trajectory cluster. The total number of the family reads per season contributing to each cluster is reported in Table S10. The pollen family seasonal contributions varied with the cluster advection pattern, likely for the contribution of aged and/or long-range transported pollen fragments. Arecaceae family contribution was largest in summer during Wslow airflows and in winter during NW airflows. Oleaceae contributions were largest in spring during Wslow and NW airflows and in winter during NE airflows. The Poaceae contribution was largest in summer during Wslow airflows and in spring during NW airflows. However, we must be aware that the small number of the collected samples during the sampling time of this study may also have contributed to the above results.

#### 3.2.2. Similarities and Dissimilarities between Lecce and Brindisi Samples

A short summary to highlight similarities and dissimilarities between the taxonomic structure of pollen samples at both sites, taking also into account the impacts of meteorological parameters and long-range transported air masses, is provided in this subsection. It has been shown in Section 3.1.2 that CR reached the largest values in Brindisi during the S3 (14 mm) and S34 (12.8 mm) samplings (Table 1) and that only three and one Urticaceae pollen grains were detected in S3 and S34, respectively. Since rain generally washes the pollen away and RH commonly increases with CR, both these meteorological parameters favoured the pollen swelling and/or rupture and, therefore, likely contributed to this result. CR also reached high values in Lecce during the S3 (10.8 mm), S34 (8.8 mm), and S12 (15.2 mm) sampling time, but no impact on the number of monitored families per sample was observed. In fact, 22, 20, and 22 pollen families were detected in S3, S34, and S12, respectively, and the number of reads was rather high (>46,000) in each of the above samples (Table S8). This result likely indicates that, in contrast to what happened in Brindisi, high RH and CR values did not significantly affect the pollen sampling in Lecce. The largest number of pollen grains was detected in Brindisi in sample S13, likely because the advection pattern (Figure S3b) favoured the advection of Oleaceae pollen, as discussed in Section 3.1.2. The pollen family read number also reached in Lecce the highest value (95,661 reads) in sample S13 (Table 4) and the read number due to Oleaceae pollen reached one of the highest values in the same sample. The advection pattern during the S13 sampling (Figure S3b) was rather similar at both sites. Note also that the largest number of pollen families was detected in Brindisi in the 24-h sample S11, which was collected in 2019 on the 9th of May, the flowering month of many pollen families (Figure S1). All pollen families (24) have also been detected in Lecce in sample S11.

Confining our analysis to the nine families shared at both sites, Figure 9 shows that Oleaceae, Poaceae, and Chenopodiaceae were among the most abundant families at both sites. Moreover, these nine families were detected in Brindisi mainly during their blooming time, while they were identified in Lecce during the whole sampling period and in all samples (Figure S6), likely for the contribution of aged and/or long-range transported pollen fragments, as mentioned. The pollen grain deposition markedly decreases with time and increasing distance from the source because of the large size of whole pollen (e.g., [47]). Consequently, the long-range transport of pollen fragments is likely favoured over that of whole pollen grains. The differences in the sampling/detection system used at both sites may have also contributed: broken pollen is not detected and, therefore, likely not collected in Brindisi. Table 5 shows, for each season, the number of reads of every pollen family detected in Lecce and shared with Brindisi. The corresponding numbers of pollen grains for the same families detected in Brindisi are given in parentheses. The nine shared pollen families were detected in each cluster and in all seasons in Lecce (Figure 13), but not in Brindisi (Figure 7). Arecaceae, Euphorbiaceae, and Poaceae were the only pollen families that reached larger contributions in the same season at both sites. However, the different sampling/detection systems used in Brindisi and Lecce have likely contributed to the results above outlined, as previously mentioned.

## 4. Summary and Conclusions

Pollen families collected in Brindisi from July 2018 to June 2019 by a Hirst-type trap and morphologically identified by optical microscopy have been compared with the corresponding ones identified by 18S rRNA gene partial sequencing in PM10 samples simultaneously collected in Lecce. Performing an exploratory study to identify airborne pollen families at both sites and discuss the benefits and limits of both sampling/detection systems represented one of the paper’s main aims. Meteorological parameters and long-range airflows, which were rather similar in Brindisi and Lecce, being 37 km apart in a beeline, and the good correlation between the pollen grain number >10 retrieved in Brindisi by optical microscopy and the pollen read number retrieved in Lecce by the DNA-metabarcoding approach have supported the comparative analyses of this study. Main results have indicated that the differences in the taxonomic structure of the samples collected in Brindisi and Lecce were mainly due to the different sampling/detection systems used at both sites, as outlined in the following.

- Whole pollen grains, whose diameter ranges from about 10 to over 100 μm, are required for the pollen morphological detection by optical microscopy in Brindisi. Indeed, the used Hirst-type impactor allows collecting only pollen from a certain equivalent aerodynamic diameter, which cannot adapt to the induced air direction changes. Conversely, the PM10 sampler used in Lecce allows the collection of only airborne pollen grains and/or fragments with an aerodynamic diameter ≤10 μm. The deposition of whole pollen grains markedly decreases with increasing distance from the source because of their large size and, consequently, the long-range transport of pollen fragments ≤10 μm and/or cytoplasmic components is likely favoured over that of pollen grains. The residence time of pollen fragments and/or cytoplasmic contents in the atmosphere may also be longer than that of whole pollen grains. Hence, pollen families were detected in Brindisi mainly during their blooming period, while they were on average detected throughout the sampling time in Lecce. Note that the health impact of pollen fragments may be more dangerous than full-size pollen grains since the former can more easily penetrate the lower respiratory tract.
- Twenty-one pollen families were detected in Brindisi throughout the sampling period of this study and pollen grains were mainly correlated with temperature. In particular, no significant correlations with wind speed were observed, likely for the use of a Hirst-type trap to collect whole pollen grains and/or for the small number of samples collected during the one-year sampling. On the contrary, five out of the twenty-four pollen families detected in Lecce were positively correlated with WS.
- Few Urticaceae pollen grains were detected in Brindisi, only during heavy rainy days, also characterised by high RH values. Pollen grains may absorb moisture from the air and swell. Consequently, especially after heavy rain, swelling may cause the grains to rupture, preventing their identification by optical microscopy. No impact of heavy rain and/or high RH on the number of detected families and reads was observed in Lecce.
- Nine out of the twenty-four Strepthophyta pollen families detected in Lecce were in common with the twenty-one pollen families detected in Brindisi. The nine families were detected in all samples in Lecce, whereas they were detected mainly in spring in the samples collected in Brindisi.
- The four-day analytical back-trajectory analysis has shown that both sites were similarly affected by airflows, but their impact on the nine shared pollen family contributions varied differently with advection patterns at both sites.

In conclusion, the paper’s results have shown that the differences between the taxonomic structure of the samples collected in Brindisi and of the corresponding ones collected in Lecce were likely due to the different pollen sampling/detection system used at both sites, which might have also been responsible for the different relationships with the meteorological parameters and long-range-transported air masses found at the two analysed sites. The main limits of the Hirst-type impactor have been highlighted. The paper’s results also suggest that the use of TSP (Total Suspended Particles) sampling heads, which allow collecting the total suspended bioaerosol particles, would be preferable to maximise the pollen collection in Lecce, with respect to e.g., PM2.5 and PM1 sampling heads, which allow collecting particles with aerodynamic diameter ≤2.5 and 1.0 μm, respectively, detecting the respirable fraction of pollen fragments.

We are aware that it would have been preferable to carry out the pollen samplings at the same monitoring site, but this was not possible for technical reasons. However, we believe that this work has provided new insights on how to organise future monitoring campaigns. The paper’s results could be of general interest to optimise pollen grain sampling/detection systems, since aeroallergens are nowadays studied all over the world, as allergic diseases are amongst the most common chronic disorders. These studies could also be helpful to public authorities in planning green spaces, so as to avoid the increase of plants that are potentially allergenic and, in this way, improve citizens’ lives.

## Figures and Tables

**Table 1:** Sampling Date (day/month/year) and duration (expressed in h) of Sampling Time (ST) interval for the 37 samples collected at Brindisi. Samples were collected in different seasons (winter: S1–S8; spring: S9–S15; summer: S16–S20; autumn: S21–S37). The sampling time started at 00.00 UTC of the Sampling Date. N° Tot. Pollen Grains shows for each sample the number of pollen grains associated with the families monitored at Brindisi according to AIA protocol, in addition to the number of pollen grains due to *Others* and *Unclassified*. The n° Families show the number of pollen families per sample monitored at Brindisi according to AIA protocol. The Shannon (*H*) and Simpson (*D*) index values at the family level are also reported for each sample. Samples are listed according to the sampling month by taking S1 collected on 7 January 2019 as the first sample.

Sample	Sampling Date (dd/mm/yy)	ST (h)	N° Tot. Pollen Grains	n° Families	At Family-Level	
					Shannon Index (*H*)	Simpson Index (*D*)
S1	07/01/2019	24	4	2	0.69	0.13
S2	17/01/2019	24	7	4	1.20	0.24
S3	24/01/2019	24	4	1	0.22	0.56
S4	31/01/2019	24	47	3	0.23	0.88
S5	07/02/2019	24	17	2	0.72	0.39
S6	14/02/2019	24	85	3	0.14	0.93
S7	21/02/2019	24	144	4	0.23	0.89
S8	28/02/2019	24	166	5	0.65	0.70
S9	04/04/2019	24	36	10	1.83	0.09
S10	18/04/2019	24	186	11	1.69	0.22
S11	09/05/2019	24	150	13	1.98	0.11
S12	16/05/2019	24	62	10	1.49	0.28
S13	23/05/2019	24	498	12	0.93	0.57
S14	30/05/2019	24	259	11	1.12	0.50
S15	06/06/2019	24	181	9	1.66	0.21
S16	04/07/2018	48	81	12	1.54	0.24
S17	11/07/2018	48	82	10	1.32	0.36
S18	18/07/2018	48	119	7	0.68	0.61
S19	25/07/2018	48	140	6	0.57	0.71
S20	19/09/2018	48	28	4	1.25	0.27
S21	03/10/2018	48	10	2	0.54	0.37
S22	10/10/2018	48	15	5	1.13	0.07
S23	17/10/2018	48	13	3	0.75	0.22
S24	24/10/2018	48	12	6	1.40	0.15
S25	07/11/2018	48	22	4	1.17	0.21
S26	12/11/2018	24	9	2	0.44	0.62
S27	14/11/2018	48	7	2	0.60	0.35
S28	21/11/2018	48	4	2	0.69	0.13
S29	28/11/2018	48	7	3	0.83	0.06
S30	03/12/2018	24	13	3	0.87	0.35
S31	10/12/2018	24	6	3	0.96	0.17
S32	11/12/2018	24	6	2	0.66	0.14
S33	12/12/2018	24	5	2	0.69	0.20
S34	17/12/2018	24	4	1	0.35	0.06
S35	18/12/2018	24	4	2	0.69	0.13
S36	19/12/2018	24	8	2	0.69	0.13
S37	20/12/2018	24	8	3	0.95	0.14

**Table 2:** Spearman correlation coefficients (reported in brackets) between meteorological parameters and pollen grain concentrations of the five families detected in more than 30% of samples, *Others* and *Unclassified* pollen grain concentrations, and the total pollen grain number (n° PGs) concentrations of the samples collected in Brindisi. Spearman correlation coefficients (reported in brackets) between meteorological parameters and the read concentrations for the nine families detected in Lecce and shared with Brindisi are also provided. The nine families shared between Lecce and Brindisi are underlined.

Meteorological Parameters	Brindisi	Lecce
	Significant Correlations	Significant Correlations
Temperature (T)	Oleaceae (0.46 **), Pinaceae (0.39 *), Poaceae (0.69 **), Urticaceae (0.60 **), *Others* (0.40 *), n° PGs (0.38 *); Cupressaceae/Taxaceae (**−0.36** *)	Poaceae (0.49 **); Asteraceae (**−0.49** **), Euphorbiaceae (**−0.40** *)
Relative Humidity (RH)		WS (**−0.36** *)
Pressure (P)	Cupressaceae/Taxaceae (0.38 *); Pinaceae (**−0.33** *)	Oleaceae (**−0.40** *)
Wind Speed (WS)		Fabaceae (0.44 **), Poaceae (0.36 *), Salicaceae (0.36 *); RH (**−0.36** *)

* p-level < 0.05; ** *p*-level < 0.01; negative correlation coefficients are in bold.

**Table 3:** Mean values ± the standard error of the mean (SEM) and minimum (Min) and maximum (Max) values of number of families, pollen grains, *Others* and *Unclassified* pollen grains, and Shannon and Simpson indices at the family level for the three main advection patterns identified in Brindisi.

Cluster		n° Families	n° Pollen Grains	n° *Others* and *Unclassified* Grains	At Family Level	
					Shannon Index (*H*)	Simpson Index (*D*)
Wslow	mean ± SEM Min–Max	6 ± 1	71 ± 26	6 ± 1	0.99 ± 0.13	0.34 ± 0.05
		1–13	1–477	1–21	0.22–1.98	0.06–0.89
NE	mean ± SEM Min–Max	5 ± 2	68 ± 36	6 ± 1	0.97 ± 0.20	0.32 ± 0.11
		2–11	8–175	2–11	0.56–1.69	0.07–0.71
NW	mean ± SEM Min–Max	4 ± 1	64 ± 30	2 ± 1	0.78 ± 0.14	0.43 ± 0.11
		2–11	3–252	0–7	0.14–1.40	0.14–0.93

**Table 4:** Number of Streptophyta reads (also considering the *Unclassified* contribution) and families for the 37 PM10 samples collected in Lecce. Shannon and Simpson indices at the family level are also reported. Samples were collected in different seasons (winter: S1–S8, spring: S9–S15, summer: S16–S20, and autumn: S21–S37). Samples are listed according to the sampling month by taking as S1 the sample collected on 7 January 2019.

Sample	n° Reads	n° Families	At Family Level	
			Shannon Index (*H*)	Simpson Index (*D*)
S1	131,194	22	1.19	0.02
S2	66,216	24	1.15	0.02
S3	173,734	22	1.10	0.03
S4	93,386	22	1.09	0.03
S5	126,821	23	1.03	0.03
S6	63,108	23	1.16	0.03
S7	150,100	23	0.71	0.12
S8	194,119	24	0.75	0.11
S9	162,986	23	1.05	0.03
S10	99,720	22	1.05	0.02
S11	142,200	24	0.96	0.03
S12	142,710	22	0.98	0.02
S13	289,789	22	0.92	0.03
S14	160,150	22	0.89	0.04
S15	227,631	22	0.90	0.04
S16	157,587	24	0.93	0.04
S17	182,051	24	1.18	0.02
S18	165,257	24	1.09	0.02
S19	141,325	23	1.02	0.03
S20	183,389	24	1.13	0.02
S21	255,624	23	1.11	0.02
S22	83,919	22	1.02	0.02
S23	108,401	22	1.07	0.02
S24	214,694	23	1.13	0.02
S25	219,449	23	1.08	0.02
S26	77,391	21	1.01	0.03
S27	197,508	22	1.09	0.02
S28	78,969	19	0.85	0.06
S29	148,741	22	1.12	0.02
S30	70,326	22	1.05	0.04
S31	204,868	23	1.12	0.03
S32	94,173	23	1.04	0.04
S33	68,166	21	1.07	0.01
S34	141,111	20	1.05	0.04
S35	101,357	22	1.17	0.02
S36	47,913	23	1.18	0.02
S37	63,885	24	1.06	0.03

**Table 5:** Total read number of the nine Streptophyta families detected in Lecce across the four seasons and shared with Brindisi dataset, and the corresponding total pollen grain number of the same nine families detected in Brindisi (reported in brackets).

Families	Winter	Spring	Summer	Autumn
Apiaceae	4473 (0)	3171 (5)	6684 (2)	10,500 (1)
Arecaceae	240 (0)	195 (0)	729 (1)	948 (0)
Asteraceae	2306 (0)	1749 (0)	640 (14)	4111 (2)
Chenopodiaceae	31,845 (0)	18,390 (75)	24,312 (13)	123,099 (2)
Euphorbiaceae	177 (3)	87 (1)	33 (0)	150 (3)
Fabaceae	10,163 (0)	11,974 (7)	24,523 (0)	46,467 (0)
Oleaceae	48,879 (0)	138,558 (675)	34,392 (6)	158,334 (8)
Poaceae	23,849 (2)	131,207 (64)	82,341 (39)	119,146 (3)
Salicaceae	912 (9)	1407 (1)	1662 (0)	894 (0)

## Data Availability

Data are contained within the article or Supplementary Material.

## Supplementary Materials

The following supporting information can be downloaded at: https://www.mdpi.com/article/10.3390/ijerph19052624/s1, Figure S1: Pollen calendar in Brindisi (2013–2015); Figure S2: Relative percentage contribution of the pollen families in Brindisi; Figure S3: Analytical back-trajectories reaching Brindisi on 9 and 23 May 2019; Figure S4: Relative abundances of pollen families associated with the airflow clusters in Brindisi; Figure S5: Relative percentage contribution of the Streptophyta families in Lecce; Figure S6: Relative percentage contribution of the pollen families in common between the two study sites; Table S1: Summary table of sampling times and meteorological parameters at both sites; Table S2: Seasonal values of meteorological parameters at both sites; Table S3: Pollen grain number of the allergenic families monitored in Brindisi; Table S4: Heat map of the within-sample relative abundances for the allergenic families in Brindisi; Table S5: Spearman’s correlation coefficients between allergenic family pollen grains and meteorological data in Brindisi samples; Table S6: Significant correlations between allergenic family pollen grains in Brindisi samples; Table S7: Total number of allergenic family pollen grains for the airflow clusters in Brindisi across the seasons; Table S8: Read number of the Streptophyta families monitored in Lecce; Table S9: Spearman’s correlation coefficients between Streptophyta family reads and meteorological data in Lecce samples; Table S10: Total read number of the families in common between the two sites for the airflow clusters in Lecce across the seasons.
